# Patterns of the Demographics, Clinical Characteristics, and Resource Utilization Among Maternal Decedents in Texas, 2001 - 2010: A Population-Based Cohort Study

**DOI:** 10.14740/jocmr2338w

**Published:** 2015-10-23

**Authors:** Lavi Oud

**Affiliations:** Division of Pulmonary and Critical Care Medicine, Department of Internal Medicine, Texas Tech University Health Sciences Center at the Permian Basin, Odessa, TX 79763, USA. Email: lavi.oud@ttuhsc.edu

**Keywords:** Mortality, Pregnancy, Resource utilization

## Abstract

**Background:**

Contemporary reporting of maternal mortality is focused on single, mutually exclusive causes of death among a minority of maternal decedents (pregnancy-related deaths), reflecting initial events leading to death. Although obstetric patients are susceptible to the lethal effects of downstream, more proximate contributors to death and to conditions not caused or precipitated by pregnancy, the burden of both categories and related patients’ attributes is invisible to clinicians and healthcare policy makers with the current reporting system. Thus, the population-level demographics, clinical characteristics, and resource utilization associated with pregnancy-associated deaths in the United States have not been adequately characterized.

**Methods:**

We used the Texas Inpatient Public Use Data File to perform a population-based cohort study of the patterns of demographics, chronic comorbidity, occurrence of early maternal demise, potential contributors to maternal death, and resource utilization among maternal decedents in the state during 2001 - 2010.

**Results:**

There were 557 maternal decedents during study period. Chronic comorbidity was reported in 45.2%. Most women (74.1%) were admitted to an ICU. Hemorrhage (27.8%), sepsis (23.5%), and cardiovascular conditions (22.6%) were the most commonly reported potential contributing conditions to maternal death, varying across categories of pregnancy-associated hospitalizations. More than one condition was reported in 39% of decedents. One in three women died during their first day of hospitalization, with no significant change over the past decade. The mean hospital length of stay was 7.9 days and total hospital charges were $250,000 or higher in 65 (11.7%) women.

**Conclusions:**

The findings of the high burden of chronic illness, patterns of occurrence of a broad array of potential contributing conditions to pregnancy-associated death, and the resource-intensive needs of a large contemporary population-based cohort of maternal decedents may better inform preventive and intervention measures at the bedside and as healthcare policy priorities. The prevalent and unchanged occurrence of rapid maternal demise following presentation for hospitalization supports a special focus on means to identify and effectively address front-line clinician- and healthcare system-related performance areas that can improve maternal outcomes. The common reporting of more than one potential contributing condition underscores the complexity of determination of causes of maternal death.

## Introduction

Maternal death is rare in high-income countries. In the United States (US), determination of the causes of maternal death is based primarily on review of death certificates, as part of the Pregnancy Mortality Surveillance System conducted by the Center of Disease Control and Preventions since 1986 [1, 2]. The Program reports on causes of pregnancy-related death, that is, “a death of a woman while pregnant or within 1 year of pregnancy termination from any cause related to or aggravated by the pregnancy or its management, but not from accidental or incidental causes” [3]. This group is a subcategory of pregnancy-associated deaths, defined as the death of a woman, from any cause, while she is pregnant or within 1 year of termination of pregnancy. While forming a practical basis for national vital statistics, determination of an underlying single cause of death through death certificates is limited by well-described inaccuracies [4], which may lead to misinformed healthcare policy and research [5-7]. In addition, determination of an association of death with pregnancy in the US depends on use of a specific checkbox on a death certificate to indicate that a decedent may have been pregnant up to 1 year prior to death. However, while clearly an improvement, a recent study by Horon and colleagues found that only 65% of pregnancy-associated deaths were captured through use of check boxes on death certificates in Maryland [8].

As importantly, death certificate-based reports focus on a single cause of death due to an underlying disease which has initiated a chain of events leading directly or indirectly to death [9]. However, this reported causal information on maternal deaths lacks the broader clinical context available in medical records on conditions both in the chain of events leading to death or those that may have contributed separately to maternal demise. Indeed, previous studies documented a low level of agreement between death certificate-based cause of death and the sequence of events described in medical records [10, 11]. In addition, despite serial cause-of-death tracking, the rate of pregnancy-related mortality has been progressively rising in the US [2], with many, if not most, events considered preventable [12-14]. On direct examination of maternal deaths in the United Kingdom (UK) by the Confidential Enquiry into Maternal Deaths, substandard care of maternal decedents was prevalent, at times with rapid deterioration and death, allowing stakeholders to make specific recommendations that may reduce maternal death [12]. A more recent report by Main and colleagues noted that 41% of pregnancy-related deaths in California had “good-to-strong” chance to be prevented, with 90% having at least “some chance” [13]. With the multisystem physiological changes associated with the demands pregnancy, the risk of further deterioration may be increased in obstetric patients with decompensated new or chronic illness, even when not directly related to or aggravated by pregnancy. For example, obstetric patients remain susceptible to the lethal effects of sepsis due to pneumonia (that is, a non-obstetric cause) or sepsis complicating well-controlled obstetric hemorrhage (i.e., sepsis as a downstream event). However, with the current reporting system on maternal death, these categories of events remain invisible to clinicians, researchers and healthcare policy makers. Thus, broader characterization of the scope of co-existing or evolving conditions among maternal decedents may better inform healthcare policy and clinical practice to identify areas for intervention needed to effectively disrupt the chain of events leading to death. However, there are presently no population-level reports, to our knowledge, on clinical patient data of maternal decedents, beyond key causes-of-death and their change over time.

We sought to characterize the patterns of the demographics, burden of chronic comorbidity, occurrence of early maternal demise, reported potential contributors to maternal death, and use of healthcare resources during terminal pregnancy-associated hospitalizations in the state of Texas during the first decade of the 21st century.

## Materials and Methods

### Setting and data sources

We used the Texas Inpatient Public Use Data File (TIPUDF), a longitudinal dataset maintained by the Texas Department of State Health Services [15], to perform a retrospective, population-based cohort study of pregnancy-associated mortality in the state. The dataset includes detailed de-identified inpatient discharge data from all state-licensed hospitals, with the exception of those exempt by state statute from reporting to the Texas Health Care Information Collection. Exempt hospitals include 1) those that do not seek insurance payment or government reimbursement and 2) selected rural providers, based on bed number and local county population. The facilities included in the mandated report account for 93-97% of all hospital discharges. The TIPUDF dataset includes demographic, clinical, resource utilization, and outcome information. The dataset includes up to 25 discharge diagnoses, and up to 25 procedures, coded using the International Classification of Diseases, Ninth Revision, Clinical Modification (ICD-9-CM). Because we used a publicly available, de-identified dataset, this study was determined to be exempt from formal review by the Texas Tech Health Sciences Center Institutional Review Board.

### Study population

We used ICD-9-CM codes (Supplementary Table 1, www.jocmr.org) to identify Texas residents with pregnancy-associated hospitalizations between 2001 and 2010. Pregnancy-associated hospitalizations with reported hospital death formed the primary cohort for the present study.

### Data collection

We collected data on patients’ age, race/ethnicity (categorized as non-Hispanic black (black), non-Hispanic white (white), Hispanic, and other), health insurance (categorized as private, Medicaid, Medicare, uninsured, and other), chronic co-morbid conditions (based on the Deyo modification of the Charlson Comorbidity Index [16]), obesity, smoking, drug and alcohol abuse, and type of pregnancy-associated hospitalization. Because of the limitations of administrative data, we have examined different categories of pregnancy-associated hospitalizations as surrogates for both phases of pregnancy and pregnancy outcomes. We categorized the type of pregnancy-associated hospitalizations into the following mutually exclusive, hierarchical groups, using pregnancy-associated ICD-9-CM codes: 1) miscarriage (pregnancies with abortive outcome, excluding induced abortion); 2) induced abortion; 3) delivery (based on the approach described by Kuklina et al [17]); 4) post-partum (hospitalizations with an ICD-9-CM code for puerperal complications, without pregnancy-related diagnosis codes of groups 1-3); and 5) antepartum (hospitalization with pregnancy-related diagnosis codes, but without those pregnancy-related diagnosis codes included in groups 1-4). We then examined the cohort of maternal decedents for reporting of specific systemic conditions, diseases, or complications of therapy, modeled on the reported categories by the pregnancy mortality surveillance system as causes of maternal death [2]. These conditions included primary or secondary diagnoses of: hemorrhage, embolism, preeclampsia/eclampsia, cardiovascular conditions, cardiomyopathy, cerebrovascular accident, sepsis, and anesthesia complications (Supplementary Table 2, www.jocmr.org). We selected sepsis, rather than infections, as a potential contributing condition to maternal death because it is the dysregulated systemic response to infection, often with resultant organ failure that is the key driver of patients’ morbidity and mortality [18, 19]. Moreover, when causes of maternal death were directly examined, it was sepsis, rather than infection, that was considered causal [12]. Similarly, sepsis, rather than infection, has been examined among causes of severe maternal morbidity [20]. The case definition of sepsis was modeled on the coding system reported by Lagu et al [21]. Finally, we collected data on decedents’ admission to an intensive care unit (ICU) (defined as presence of an ICU charge greater than $0), hospital length of stay and total hospital charges.

### Data analysis

We report on pregnancy-associated deaths, that is, focusing on decedents of pregnancy-associated hospitalizations, without determination whether death has been due to any cause related to or aggravated by the pregnancy or its management [3]. Because administrative datasets preclude assessment of causality of reported clinical conditions or procedures, we examined reporting of each category of the aforementioned causes of maternal death as *potential contributing conditions* (termed similarly in the remainder of the manuscript) among maternal decedents. However, while presence of the examined potential contributing conditions is unlikely to be inconsequential, the magnitude of the contribution of each could not be inferred. Rather, we describe pregnancy-associated deaths *with* any of the examined potential contributing conditions. We examined the distribution of potential contributing conditions for the whole cohort and across categories of pregnancy-associated hospitalizations, in line with prior national reports on pregnancy-related mortality across pregnancy outcomes [2]. Because only four maternal decedents had reported anesthesia complications, no further subgroup examination was carried out on these events. Although some of the examined chronic comorbidities may have overlapped with the potentially contributing conditions, and others may have contributed to maternal death, the primary focus of the present study was reporting of the potentially contributing conditions as defined earlier.

Eleven hospitalizations associated with miscarriage/induced abortion could not be adequately classified to only one group (that is, either miscarriage or induced abortion), because their only pregnancy-associated ICD-9-CM code was 639.XX (complications following abortion and ectopic and molar pregnancies). We thus combined hospitalizations associated with miscarriage and induced abortion into a single group.

Since changes over time have been reported in the rates of specific causes of pregnancy-related deaths, we further reviewed changes over time in reporting of the examined potential contributing conditions between 2001 - 2005 and 2006 - 2010, similar to prior reports [2].

Because timely recognition and prompt effective interventions have been recognized as key factors to limit preventable maternal death [12, 13], events of rapid deterioration and death can serve as a unique focus area for review in order to better clinical practice. Because administrative datasets do not provide information on the time to onset of potential contributing conditions, and specifically from start of deterioration to maternal death during hospital course, we performed a subgroup examination of maternal decedents who died during the first day of hospitalization. This evaluation was carried out in order to examine the contemporary scope of early rapid maternal demise following presentation to a hospital and to characterize the demographic and clinical attributes of this subgroup. We then examined each of these attributes in the subgroup as share of the whole cohort.

We used data on the demographic characteristics, health insurance, and category of hospitalization of all pregnancy-associated hospitalizations (Supplementary Table 3, www.jocmr.org) as contemporaneous background information for examination of the relative representation of the corresponding attributes among maternal decedents.

Total hospital charges were examined following standardization to 2010 US dollars, using the annual consumer price index [22].

Group data are reported as numbers (percentages) for categorical variables and mean (standard deviation (SD)) or median (interquartile range (IQR)) for continuous variables, as appropriate. Distribution of normality was examined by Kolmogorov-Smirnov test. Categorical data were compared by a two-sided X^2^ test. All statistical analyses were performed using MedCalc version 15.6 (MedCalc Software, Ostend, Belgium) and SAS version 9.3 (SAS Institute, Cary, NC, USA). A two-sided P value < 0.05 was considered significant.

## Results

There were 4,060,659 pregnancy-associated hospitalizations, with 557 hospital deaths during the 2001 - 2010 period. The demographic characteristics, type of health insurance and category of pregnancy-associated hospitalization of maternal decedents are detailed in Table 1. The distribution of age, race/ethnicity, health insurance, and categories of pregnancy-associated hospitalization among maternal decedents varied considerably, as compared to that among all pregnancy-associated hospitalizations. Thus, age of 35 years or older, black race, and lack of health insurance occurred each at rates nearly two-fold higher among decedents, with corresponding lower rates among younger women, and those of Hispanic and white race/ethnicity. When considering the category of pregnancy-associated hospitalization, delivery hospitalizations were markedly underrepresented among decedents, while the rates of postpartum, abortion/miscarriage, and antepartum hospitalizations were about 20-fold, six-fold, and two-fold higher, respectively, as compared to their distribution among all pregnancy-associated hospitalizations.

**Table 1:** The Demographic Characteristics, Health Insurance and Categories of Pregnancy-Associated Hospitalizations Among Maternal Decedents

Group	Maternal decedents (n = 557)
Age (years), n (%)	
< 18	22 (3.9)
18 - 34	411 (73.8)
≥ 35	124 (22.3)
Race/ethnicity, n (%)	
Hispanic	227 (40.8)
Black	127 (22.8)
White	156 (28)
Other	47 (8.4)
Health insurance, n (%)	
Private	169 (30.3)
Medicaid	268 (48.1)
Medicare	16 (2.9)
Uninsured	88 (15.8)
Other	16 (2.9)
Category of hospitalization, n (%)	
Miscarriage/abortion	61 (11)
Delivery	321 (57.6)
Postpartum	100 (18)
Antepartum	75 (13.4)

The chronic co-morbidities of maternal decedents are detailed in Table 2. One or more chronic co-morbidity was reported in 45.2%, with the most common being cerebrovascular disease (13.8%), chronic liver disease (11.3%), and congestive heart failure (9.5%). Obesity, smoking, ethanol or drug abuse were rarely reported.

**Table 2:** Chronic Comorbidities Among Maternal Decedents

Group	Maternal decedents (n = 557)
Any chronic comorbidity, n (%)^1^	252 (45.2)
Deyo-Charlson score	
Median (IQR)	0 (0 - 1)
Mean (SD)	0.9 (1.5)
Myocardial infarction	19 (3.4)
Congestive heart failure	53 (9.5)
Peripheral vascular disease	26 (4.7)
Cerebrovascular disease	77 (13.8)
Chronic lung disease	24 (4.3)
Connective tissue disease	16 (2.9)
Diabetes mellitus	27 (4.8)
Chronic kidney disease	15 (2.7)
Malignancy	17 (3.1)
Chronic liver disease	63 (11.3)
Human immunodeficiency virus infection	9 (1.6)
Obesity	24 (4.3)
Alcohol abuse	1 (0.2)
Drug abuse	32 (5.7)
Smoking	7 (1.3)

^1^Based on conditions included in the Deyo-Charlson comorbidity index. IQR: interquartile range; SD: standard deviation.

The distribution of the examined potential contributing conditions for the whole cohort and across categories of pregnancy-associated hospitalizations is detailed in Tables 3 and 4, respectively. The most commonly reported potential contributing conditions among examined categories were hemorrhage (27.8%), sepsis (23.5%), and cardiovascular conditions (22.6%). The comparative reporting of potential contributing conditions between 2001 - 2005 and 2006 - 2010 is detailed in Supplementary Table 4 (www.jocmr.org), showing no significant change between the two periods, with the exception of sepsis, which rose from 19% to 27.1% (P = 0.0332). More than one potential contributing condition was reported in 217 (39%) decedents.

**Table 3:** Potential Contributing Conditions Among Maternal Decedents

Group	Maternal decedents (n = 557)
Hemorrhage, n (%)	155 (27.8)
Embolism, n (%)	62 (11.1)
Preeclampsia/eclampsia, n (%)	111 (19.9)
Sepsis, n (%)	131 (23.5)
Anesthesia complications, n (%)	4 (0.7)
Cardiomyopathy, n (%)	71 (12.7)
Cerebrovascular accident, n (%)	91 (16.3)
Cardiovascular conditions, n (%)	126 (22.6)

**Table 4:** Potential Contributing Conditions Among Maternal Decedents, Across Categories of Pregnancy-Associated Hospitalizations

Group	Category of pregnancy-associated hospitalization			
	Miscarriage/abortion (n = 61)	Delivery (n = 321)	Postpartum (n = 100)	Antepartum (n = 75)
Hemorrhage, n (%)^1^	19 (31.1)	124 (38.6)	3 (3)	9 (12)
Embolism, n (%)	5 (8.2)	40 (12.5)	13 (13)	4 (5.3)
Preeclampsia/eclampsia, n (%)	2 (3.3)	94 (29.3)	12 (12)	3 (4)
Sepsis, n (%)	24 (39.3)	61 (19)	29 (29)	17 (22.7)
Cardiomyopathy, n (%)	4 (6.6)	27 (8.4)	35 (35)	5 (6.7)
Cerebrovascular accident, n (%)	3 (4.9)	40 (12.5)	44 (44)	4 (5.3)
Cardiovascular conditions, n (%)	15 (24.6)	52 (16.2)	46 (46)	13 (17.3)

^1^Percentage figures refer to the frequency of reporting of the examined potential contributing condition among maternal decedents during a specific category of pregnancy-associated hospitalization.

The distribution of the potential contributing conditions varied substantially across categories of pregnancy-associated hospitalizations. Hemorrhage (38.6%) and preeclampsia/eclampsia (29.3%) were reported at disproportionately higher rates during delivery hospitalizations, while cardiovascular conditions (46%), cardiomyopathy (35%), and cerebrovascular accidents (44%) were noted during postpartum hospitalizations at rates more than two-fold higher than for the whole cohort. Sepsis was especially common among maternal decedents hospitalized in association with abortion or miscarriage (39.3%), but also during postpartum hospitalizations (29%).

One hundred ninety-two women (34.5%) died during their first day of hospitalization. The demographic and clinical characteristics of this subgroup and their respective share out of the whole cohort are described in Tables 5 and 6. No significant change occurred in the rate of death on day 1 of hospitalization between the years 2001 - 2005 and 2006 - 2010 (36.8% vs. 32.6%; P = 0.3363). When considering the occurrence of specific attributes of women who died during their first day of hospitalization, as their share of the whole cohort, women 35 years or older were increasingly represented (37.9%), as were white women (43.5%), while Hispanic ones were underrepresented (26.4%). Both uninsured women (44.3%) and those with private insurance (42%) were disproportionately represented among decedents dying on the first day of hospitalization. In addition, deaths on the first day of hospitalization occurred disproportionately more often during antepartum hospitalizations (42.7%) and those associated with abortion or miscarriage (37.7%).

**Table 5:** The Demographic Characteristics and Health Insurance Among the Women Who Died During Their First Day of Hospitalizations and the Share of the Examined Characteristics out of the Whole Cohort of Maternal Decedents

Group	Deaths on hospital day 1 (n = 192)	All maternal decedents (n = 557)
Age (years), n (%)		
< 18	7 (3.6)^1^	7/22 (31.8)^2^
18 - 34	138 (78.9)	138/411 (33.6)
≥ 35	47 (24.5)	47/124 (37.9)
Race/ethnicity, n (%)		
Hispanic	60 (31.3)	60/227 (26.4)
Black	42 (21.9)	42/127 (33)
White	68 (35.4)	68/156 (43.6)
Other	22 (11.5)	22/47 (46.8)
Health insurance, n (%)		
Private	71 (37)	71/169 (42)
Medicaid	79 (41.1)	79/268 (29.5)
Medicare	2 (1)	2/16 (12.5)
Uninsured	39 (20.3)	39/88 (44.3)
Other	1 (0.5)	1/16 (6.3)

^1^Percentages represent occurrence of examined attributes among the women who died during their first day of hospitalization. ^2^Percentages represent occurrence of the examined attributes among women who died during their first day of hospitalization out of all maternal decedents with the same attribute.

**Table 6:** The Categories of Pregnancy-Associated Hospitalizations and Potential Contributing Conditions Among the Women Who Died During Their First Day of Hospitalizations and the Share of the Examined Characteristics out of the Whole Cohort of Maternal Decedents

Group	Deaths on hospital day 1 (n = 192)	All maternal decedents (n = 557)
Category of hospitalization, n (%)		
Miscarriage/abortion	23 (12)^1^	23/61 (37.7)^2^
Delivery	114 (59.4)	114/321 (35.5)
Postpartum	23 (12)	23/100 (23)
Antepartum	32 (16.7)	32/75 (42.7)
Potential contributing conditions		
Hemorrhage, n (%)	65 (33.9)	65/153 (39.9)
Embolism, n (%)	31 (16.1)	31/62 (50)
Preeclampsia/eclampsia, n (%)	29 (15.1)	29/111 (26.1)
Sepsis, n (%)	23 (12)	23/131 (17.6)
Cardiomyopathy, n (%)	6 (3.1)	6/43 (14)
Cerebrovascular accident, n (%)	15 (7.8)	15/77 (19.5)
Cardiovascular conditions, n (%)	22 (11.5)	22/93 (23.7)

^1^Percentages represent occurrence of examined attributes among the women who died during their first day of hospitalization. ^2^Percentages represent occurrence of the examined attributes among women who died during their first day of hospitalization out of all maternal decedents with the same attribute.

The share of potential contributing conditions among women dying on their first day of hospitalization out the whole cohort varied considerably, with hemorrhage (39.9%) and embolism (50%) overrepresented, while the remaining examined conditions were reported at disproportionately lower rates.

Details of resource utilization among maternal decedents are outlined in Table 7. Admission to ICU occurred in 413 (74.1%) of all decedents and in 125 (65.1%) of those who died on their first day of hospitalization. Hospital length of stay of decedents was 14 days or longer in 82 (14.7%) decedents. Inflation-adjusted total hospital charges were $250,000 or higher in 65 (11.7%) decedents, with their mean (SD) length of stay 35.2 (38.8) days. The highest inflation-adjusted hospital charge was approximately $4,300,000.

**Table 7:** Resource Utilization Among Maternal Decedents

Group	Maternal decedents (n = 557)
Admission to ICU, n (%)	413 (74.1)
Total hospital charges (dollars)^1^	
Median (IQR)	57,709 (24,132 - 119,273)
Mean (SD)	139,029 (340,739)
Hospital length of stay (days)	
Median (IQR)	3 (1 - 8)
Mean (SD)	7.9 (17)

^1^Adjusted for inflation to 2010 dollars.

## Discussion

The major findings of the present study are: 1) nearly half of maternal decedents had one or more reported chronic co-morbidity; 2) the frequency of pregnancy-associated hospital death varied substantially across categories of pregnancy-associated hospitalization, occurring most often during postpartum hospitalizations; 3) more than one potential contributing condition was reported in 39% of women; and 4) one in three women died on their first day of hospitalization, with no significant change over the past decade.

The present study is, to our knowledge, the first population-level examination of the patterns of the demographic and clinical characteristics of maternal decedents beyond single cause-of-death reports. Although numerous studies of obstetric patients included data on maternal decedents, both through direct review of medical records [13, 23, 24] or using administrative data [25-27], the investigators focused on single causes of death of decedents [13, 23] or reported conditions associated with combined maternal morbidity and mortality [24-27], without systematic examination of overall occurrence of common potential causes of maternal death. Because we studied the contribution of the most commonly reported causes of maternal death among all pregnancy-associated deaths during women’s terminal hospitalization, our findings are not directly comparable to prior reports on maternal decedents. Finally, even when focusing exclusively on maternal decedents with use of clinical records, in a recent study by Main and colleagues on women’s characteristics and improvement opportunities in pregnancy-related mortality, the investigators reported only on selected demographics and obstetric features, with no data on chronic illness, overall occurrence of potential causes of death, or resource utilization [13].

Our findings that older and black women are disproportionately represented among pregnancy-associated deaths in the present cohort are consistent with data from national studies of pregnancy-related mortality [2]. The health insurance of maternal decedents in the US has not been previously reported at a population level, to our knowledge. Medicaid was the most common source of health insurance among maternal decedents, similar to the obstetric population in the state [28]. The underrepresentation of private insurance among decedents in the present cohort is expected and reflects previously reported better associated obstetric outcomes [29]. On the other hand, the disproportionately higher frequency of Medicare insurance among maternal decedents likely reflects an increased burden of chronic illness in this young population. Finally, lack of health insurance among maternal decedents, occurring at a rate two-fold higher than among all pregnancy-associated hospitalizations, extends prior findings of an adverse impact of lack of adequate prenatal care [29, 30] to pregnancy-associated mortality.

Chronic co-morbid conditions were common in the present cohort, reported in nearly one in two maternal decedents. While chronic illness that may have contributed to death is often included in death certificates, the reported studies on pregnancy-related mortality in the US do not include data on the burden of chronic illness beyond single cause of death [2, 13]. Cerebrovascular disease, congestive heart failure, and chronic liver disease were the most commonly reported chronic conditions in the present cohort. However, use of administrative data precludes inference about the actual duration, severity and clinical course of the reported conditions. In a recent study of pregnancy-related deaths in the UK by Nair and colleagues, the investigators reported one or more pre-existing medical problems in 68.9% of decedents [31]. However, the authors examined a different set of medical conditions (for example, mental health problems or infertility) than those included in the Deyo-Charlson comorbidity index [16], precluding direct comparison. Nevertheless, both studies suggest prevalent chronic illness among maternal decedents that may have affected their outcomes, even if not being directly causal.

Although most pregnancy-associated deaths occurred during delivery hospitalizations, death occurred more often during remainder pregnancy-associated hospitalizations. This discrepancy can be expected because most delivery hospitalizations represent routine admission among healthy women, while the remainder categories of pregnancy-associated hospitalization involved, by definition, complications of pregnancy. Nevertheless, the latter does not explain the substantial difference in frequency of maternal death across categories of non-delivery hospitalizations. Thus, pregnancy-associated deaths occurred during postpartum hospitalizations in 18% of decedents, although postpartum hospitalizations accounted for less than 1% of all pregnancy-associated hospitalizations. The latter finding indicates that in the present cohort women were most likely to die during postpartum hospitalization. Recent data indicate that postpartum hospitalizations are associated with much higher risk of severe maternal morbidity than delivery hospitalizations. In a study by Callaghan and colleagues, the investigators found in a national US sample that severe maternal morbidity occurred at a 14-fold higher rate among postpartum hospitalizations than among delivery hospitalizations [32]. The latter findings may explain the high frequency of pregnancy-associated deaths during postpartum hospitalizations. There are, however, no corresponding comparative studies, to our knowledge, on antepartum hospitalizations or those associated with abortion or miscarriage. Thus, the sources of the differential frequency of pregnancy-associated death observed across non-delivery hospitalizations are uncertain. Although it can be hypothesized that the varying occurrence of potential contributing conditions and other patient factors may have had different adverse impact during different phases of pregnancy, our study design precludes assessment of causal role of specific patient attributes and, as importantly, does not allow adequate assessment of possible differences in patient care and healthcare system contributions during different categories of pregnancy-associated hospitalizations.

We found that hemorrhage, sepsis, and cardiovascular conditions were the most commonly reported potential contributing conditions among pregnancy-associated hospital deaths, with the frequency of their reporting markedly higher, than that among pregnancy-related deaths [2]. There was no significant change in frequency of the examined potential contributing conditions between the years 2001 - 2005 and 2006 - 2010, with the exception of sepsis. The latter findings appear to contrast recent reports on decreasing pregnancy-related mortality due to hemorrhage, preeclampsia/eclampsia, embolism and the rising mortality due to cardiovascular conditions and cardiomyopathy [2]. Because we examined reporting of potential contributing conditions, rather than causes of death, the findings of higher frequency of the potentially contributing conditions among maternal decedents than that among pregnancy-related deaths are to be expected. Similarly, trends of the occurrence of potential contributing conditions over time may not necessarily match their role as a single cause of death in the subset of pregnancy-related mortality. Our finding of increased reporting of sepsis among maternal decedents over time complements the study by the Confidential Enquiry on Maternal Deaths, showing on direct examination of clinical records a rising mortality rate due to sepsis, making it the most common cause of direct maternal death in the UK [12]. In addition, recent population-based studies in the US described a rising rate of sepsis-associated mortality in the obstetric population [33, 34]. Because we did not limit our study to a single potential cause of death, the distribution of the examined potential contributing conditions is, as expected, at variance with the frequency of reported single, mutually exclusive, causes of pregnancy-related mortality both in general and over time. The findings of the present study provide broader characterization of the occurrence of potentially life-threatening conditions that may have played either a direct causal role or as additional downstream factors contributing to women’s demise.

The distribution of most of the potential contributing conditions varied considerably across specific categories of pregnancy-associated hospitalization. Our findings are not directly comparable to national reports on pregnancy-related mortality due to our use of categories of pregnancy-associated hospitalization, rather than specific pregnancy outcomes. Thus, national reports on pregnancy-related mortality did not have data on separate postpartum hospitalizations and the outcome of pregnancy was unknown in about 10% of decedents [2]. In addition, our focus was on overall reporting on potential contributing conditions among pregnancy-associated deaths. However, we found similarly to national reports [2] that hemorrhage and infection occurred at higher rates among decedents during abortion/miscarriage-associated hospitalizations. In addition, our findings of disproportionately higher reported frequency of cardiovascular conditions, cardiomyopathy and cerebrovascular accidents among decedents during postpartum hospitalizations extend the results of population-based studies showing increased occurrence of severe maternal morbidity due to cardiac and cerebrovascular conditions during postpartum hospitalizations [35, 36].

We found, not unexpectedly, that more than one potential contributing condition was reported in 39% of maternal decedents. While causal relationship between the reported conditions and patients’ outcome could not be inferred, the latter finding underscores the challenges and limitations of determination of causes of maternal death, and those of the current single-cause- of-death reporting when striving to apply findings to bedside clinical practice.

Rapid maternal demise following presentation to inpatient care has been common in our cohort, with one in three maternal decedents dying during the first day of hospitalization, showing no significant change over the past decade. Because the time from development of conditions causing maternal deterioration to death is not available in administrative datasets, our findings likely underestimate the occurrence of rapid maternal demise. We found unexpectedly that white women were overrepresented, while minority women (Hispanics) were underrepresented among those who died on the first day of hospitalization. Nearly half of all maternal decedents without health insurance died on the first day of hospitalization, likely reflecting lack of regular care and possibly associated lack of readily available early outpatient care during evolving acute problems. However, unexpectedly, death during the first day of hospitalization occurred nearly as frequently among maternal decedents with private insurance. The sources of the observed demographic patterns in the subgroup of early hospital deaths, not previously reported, are unclear and require further study.

Deaths among maternal decedents with reported hemorrhage and embolism occurred at disproportionate higher frequently on the first day of hospitalization, suggesting common abrupt deterioration with these complications when occurring on presentation to inpatient care or shortly afterwards. On the other hand, most other potential contributing conditions were noted at disproportionately lower rate in this subgroup. However, the small number of women in various subgroups limits adequate comparative analysis of their demographic and clinical attributes.

Although the design of the present study precluded examination of the preventability of pregnancy-associated mortality among individual decedents, our findings about the women who died on their first day of hospitalization do not suggest that these early deaths are uniquely or mostly unavoidable. Rather, the finding of prevalent early maternal demise shown in the present study supports a special focus of future investigations on means to identify and effectively address front-line clinician- and health care system-related performance areas that can improve maternal outcomes. In addition, better outreach to patients, in order to address previously demonstrated remediable areas, such as delays in seeking care and lack of knowledge [13] may further assist in reducing current fatal outcomes.

Nearly three in four maternal decedents were managed in an ICU. This finding indicates that clinicians have recognized a higher risk for deterioration or an acute critical state in most women. However, the administrative data preclude determination of timeliness of ICU transfer or the appropriateness and timeliness of clinical interventions [12, 37]. Similarly, the reasons the remainder women were not admitted to ICU cannot be inferred. While there may have been early warning signs that were not recognized by clinicians, followed by rapid deterioration and death, it is conceivable that in some maternal decedents there has been abrupt, unpredictable deterioration, as may be the case with massive pulmonary embolism. Admission to an ICU was less frequent among women who died on their first day of hospitalization, suggesting that clinical deterioration may have been too rapid for many.

Early consultation with a critical care specialist and collaborative ICU care have been strongly advocated, and indicators to consider ICU transfer were outlined [12, 37]. However, in order to translate into improved maternal outcomes, these consultations should best occur within a framework of improvement in other, pre-ICU, clinician- and healthcare system-related factors [12, 13].

Only limited data are available on ICU utilization among maternal decedents. In a recent study of obstetric ICU admissions in Maryland by Wanderer and colleagues, the majority (58.6%) of pregnancy-associated deaths occurred without ICU admission [38]. The investigators hypothesized that ICU may have been underutilized in this population or that the pace of deterioration precluded ICU transfer. These findings likely reflect variability in both clinical practice and patient mix and suggest another area for future study in order to consistently assure timely ICU care.

Although a third of maternal decedents died on their first day of hospitalization, the mean hospital length of stay was three-fold longer than that for all maternal hospitalizations in the state [28], with nearly one in seven decedents having length of stay 14 days or longer, reflecting women’s substantial morbidity.

The mean total hospital charges for maternal decedents were nearly seven-fold higher than those for all maternal hospitalizations in Texas [28], and exceeded $250,000 in one in 10 women. These findings document an expected level of commitment by clinicians to a mostly healthy population experiencing an acute severe illness, who usually survive. Because timely recognition of early life-threatening events, coupled with prompt effective interventions can improve both likelihood of survival and reduce risk of downstream development of often irreversible organ failure, data on the prevalent lapses in these areas in the care of maternal decedents [12, 13] suggest that improved clinical care may prevent or at least limit the need for prolonged and often burdensome life-support interventions.

Our findings should be considered in the context of several limitations. First, the retrospective design of the present study and use of an administrative dataset with their attendant limitations affect the interpretation of our results. However, the rarity of pregnancy-associated mortality in the US remains a challenge for population-based approaches.

In addition, our cohort was based on identified pregnancy-associated hospitalizations. Thus, we may have omitted maternal deaths among hospitalizations not coded as pregnancy-associated. However, pregnancy-associated hospitalizations were the most common category of hospitalization [28] in Texas, with expected high clinician and coder familiarity. In addition, we have likely omitted hospitalizations beyond the postpartum period, but less than 1 year following end of hospitalization, and could not examine pregnancy-associated deaths occurring outside the hospital. Nevertheless, similar methodological limitations have affected other population-based studies of maternal death based on administrative data [23, 25-27], as well as studies of severe maternal morbidity [20]. Finally, the proportions of the various categories of pregnancy-associated hospitalizations in the present study were comparable to those reported by other investigators [39].

We have identified the examined potential contributing conditions based on specific ICD-9-CM codes. Thus, we cannot exclude possible misclassification. However, our approach for identification of each of the examined conditions was based on a very broad code array and is similar to that reported by other investigators [20, 25-27]. Thus, a systematic bias in the annual reporting of the examined conditions or general underestimation is unlikely.

The optimal ICD-9 code-based approach to identify patients with sepsis remains unsettled. We chose a highly sensitive and specific approach [40], used by other investigators in the general population [21, 40]. However, we cannot exclude a possibility of underestimating occurrence of sepsis hospitalizations. In addition, a recent report by Gaieski et al indicates that various approaches to identify sepsis, and specifically the severe sepsis subgroup, using administrative data in the general population demonstrate similar upward trends over time [41]. Thus, it is unlikely that the observed increased occurrence of sepsis in the present cohort is due to our case identification approach.

Finally, we examined pregnancy-associated deaths in Texas due to its large and diverse population, with a high-quality, longitudinal hospital discharge dataset. However, population characteristics, clinical practice patterns, and the occurrence of the examined potential contributing conditions among maternal decedents may vary across states and nationally, as is the case for pregnancy-related mortality [8, 13]. Further studies on the demographic and health insurance attributes, occurrence of the examined potential contributing conditions, burden of chronic illness and resource needs in pregnancy-associated mortality are warranted in other healthcare environments.

Because findings of substandard clinical care of maternal decedents, mostly related to lack of timely recognition and prompt effective intervention, were prevalent in prior investigations of maternal death [12, 13], along with identified potentially preventable adverse health system and patient factors [13], it has been suggested that a majority of maternal deaths may be preventable [12, 13]. The reported practice deficiencies are especially disconcerting, as the findings of the present study demonstrate that obstetric patients remain vulnerable to rapid, fatal deterioration which continued to occur commonly among maternal decedents. The present findings underscore the urgent need for direct systematic reviews of all maternal deaths following the lead of other high-income countries [12], in order to formulate workable measures to address clinician, health system and patient factors underlying the many preventable maternal mortality events [13, 14]. However, although detailed actionable recommendations have been recently proposed in the UK based on the Confidential Enquiry into Maternal Deaths [12], their implementation and impact remain unknown.

In summary, our findings for the whole cohort and among women who died during their first day of hospitalization demonstrate a complex interplay of the demographic and clinical attributes among maternal decedents. The varying profiles of potential contributing conditions and the other described women’s attributes observed during different categories of pregnancy-associated hospitalizations and among those dying on their first day of hospitalization may help prioritize surveillance, preventive, and early interventional efforts necessary to improve maternal outcomes.

Thus, moving beyond an exclusive focus on the frequency of single causes of death, the findings of present study complement death certificate-based reports and highlight opportunities to more effectively disrupt the chain of events leading to fatal maternal outcomes, through better informed healthcare policy and bedside practice.
